# Putrescine-Mediated Modulation of Morphological and Reproductive Differentiation During Direct In Vitro Regeneration of *Rosa chinensis* cv. Old Blush

**DOI:** 10.3390/biology15151266

**Published:** 2026-08-02

**Authors:** Arash Zahedi, Mahyar Gerami, Mahboubeh Davoudi Pahnekolayi

**Affiliations:** 1 Ornamental Plants Research Group, Department of Landscape Design, Sana Institute of Higher Education, Sari 48177, Iran; 2 Department of Biology, Sana Institute of Higher Education, Sari 48177, Iran; 3 Research Group Phytochemistry and Biochemistry of Natural Compounds, Institute of Chemical, Environmental and Biosciences Engineering, Technische Universität Wien (TU Wien), 1040 Vienna, Austria

**Keywords:** biostimulants, direct regeneration, growth additives, in vitro flowering, *Rosa Chinensis*, polyamines

## Abstract

In vitro regeneration and flowering systems provide valuable platforms for rose breeding and biotechnological applications. Although the effects of plant growth regulators (PGRs) on Rose regeneration have been extensively studied, the contribution of growth additives, either alone or in combination with PGRs, remains insufficiently understood in *Rosa chinensis*. This study investigated the effects of PGRs (6-Benzylaminopurine (BAP), 1-Naphthaleneacetic acid (NAA), and indole-butyric acid (IBA)) combined with three growth additives, putrescine (Put), phloroglucinol (PG), and casein hydrolysate (CH), at different concentrations on the vegetative and reproductive responses of *R. chinensis* in an in vitro system. No flowering occurred in media without PGRs, regardless of the growth additive treatment, highlighting the essential role of PGRs in floral induction. However, the integration of growth additives with PGRs significantly enhanced regeneration and flowering performance. Among the tested additives, Put showed the strongest stimulatory effect, increasing in vitro flower production and improving floral characteristics. PG promoted several morphological traits, including shoot regeneration and flower stem elongation, but was less effective than Put. CH exhibited limited effects on both vegetative growth and reproductive development. These findings indicate that Put supplementation in PGR-based media is an effective approach for improving regeneration and flowering efficiency in *R. chinensis*, with potential applications in rose biotechnology and breeding programs.

## Simple Summary

Efficient in vitro regeneration and flowering systems are important tools for rose breeding, conservation, and biotechnology. While plant growth regulators are widely used to promote regeneration, the potential benefits of additional growth-promoting compounds have received less attention in *Rosa chinensis*. In this study, we evaluated the effects of three growth additives—putrescine, phloroglucinol, and casein hydrolysate—used alone or in combination with plant growth regulators. Our results demonstrated that plant growth regulators were essential for inducing regeneration and flowering, as no flowers developed in their absence. Putrescine produced the greatest improvements in shoot regeneration and in vitro flowering, when combined with plant growth regulators, whereas phloroglucinol enhanced several shoot and flower quality traits. Casein hydrolysate showed comparatively limited benefits under the tested conditions. These findings demonstrate that supplementing conventional regeneration media with putrescine can improve the efficiency of in vitro propagation and flowering of *Rosa chinensis*. The results provide practical information for developing improved micropropagation protocols that may support future rose breeding and biotechnological applications.

## 1. Introduction

Roses are precious ornamental plants that belong to the genus *Rosa* in the Rosaceae family. They are used as cut flowers, potted plants, and for gardens and landscaping. Additionally, they play a vital role in various industries, including food, cosmetics, pharmacology, and medicine [1]. The *Rosa* genus includes over 200 species, with *Rosa chinensis* being one of the most significant in the history of rose cultivation. Their popularity originates from their ability to bloom continuously throughout the year, which significantly increases their demand [2,3]. Therefore, employing plant biotechnology approaches and in vitro culture techniques can enhance their production in terms of quality and quantity, while also providing benefits for genetic improvement and genome preservation [4]. Therefore, establishing efficient in vitro propagation is recommended.

On the other hand, despite the diversity and wide range of flower colors in roses, floral trait improvement remains a primary breeding approach in rose breeding programs [5]. Given the lengthy timelines, high levels of genetic heterozygosity, and varying ploidy levels in traditional breeding methods, molecular breeding offers a technical approach to precise modification of roses [3]. In recent years, molecular breeding techniques have been widely used to improve flower color in roses, primarily through tissue culture [6,7,8]. Therefore, molecular breeding of roses for various objectives is feasible, and it is highly dependent on the establishment of a successful in vitro regeneration protocol (organogenesis & somatic embryogenesis) to enhance efficiency.

Among various in vitro culture techniques, direct organogenesis, in particular, is a preferred regeneration pathway with the benefit of minimizing the possibility of somaclonal variations by avoiding an intervening callus phase [9,10]. The success of in vitro regeneration protocols, including direct organogenesis, is heavily dependent on responsive interactions between culture media ingredients, including Plant Growth Regulators (PGRs) and available growth-promoting adjuvants [11]. This study focuses on the effect of three specific growth additives (Putrescine (Put), Casein Hydrolysate (CH), and Phloroglucinol (PG)) with/without PGRs on direct organogenesis efficiency in *R. chinensis*.

Polyamines (PAs), such as Put, Spermidine (Spd), and Spermine (Spm), are organic compounds with important roles in the plant growth and development process [12]. PAs and their biosynthetic enzymes are involved in a wide range of metabolic events, ranging from protecting plants against stress to cell organogenesis. Their exogenous application in in vitro culture systems enhanced direct organogenesis through promoting cell division, modulating hormonal crosstalk, and alleviating oxidative stress [13,14,15,16,17]. Exogenous application of Spd in *Arabidopsis thaliana* lateral root primordia explants promoted regeneration efficiency and directly regenerated shoot meristems without an intervening callus phase [18]. Depending on the plant species and type of explants being used for in vitro culture, the optimal concentration of PAs varied [19].

PG is a relatively unknown compound with growth-promoting properties [20]. It is a phenolic compound recognized as a natural precursor of lignin biosynthesis with complex roles in plant tissue culture. It increases shoot formation and somatic embryogenesis in some horticultural crops and mainly acts as an auxin synergist to improve root induction and shoot elongation [9,21]. Moreover, it can modulate in vitro stress damage and control hyperhydricity (vitrification), a common challenge in woody plant in vitro cultures, by enhancing lignin biosynthesis pathways [22]. PG demonstrates both cytokinin-like and auxin-like activity, which makes it a potential candidate for a wide range of plant tissue culture studies [20].

CH is another complex organic additive reported to promote in vitro growth and differentiation in various crops, including callus growth and somatic embryogenesis [23]. CH is a complex organic nitrogen source rich in diverse amino acids and small peptides that are commonly employed to promote plant cell cultures [24]. In our previous research study, the promising role of CH in enhancing regeneration efficacy was emphasized through facilitating nitrogen availability for in vitro explants during the proliferation stage of *R. canina* [11]. It has been demonstrated that plants in vitro have a greater capacity to mobilize and transfer nitrogen from organic sources than from inorganic ones [25].

Although Put, PG, and CH have each been reported to improve specific aspects of in vitro culture in several plant species, including shoot regeneration, rooting, somatic embryogenesis, and stress mitigation, their effects are highly species- and genotype-dependent. To date, limited information is available regarding their comparative effects on direct organogenesis and in vitro flowering in *Rosa chinensis*, particularly when applied individually or in combination with PGRs. Furthermore, the potential interactions between these growth additives and conventional PGR-based regeneration systems remain poorly understood. This lack of knowledge limits the optimization of efficient regeneration protocols required for genetic transformation and molecular breeding of roses.

In addition to efficient shoot regeneration, the induction of in vitro flowering has attracted increasing attention as a valuable developmental and biotechnological trait. It provides a rapid system for investigating floral induction and reproductive development under controlled conditions while reducing the time required to reach the flowering stage [6,7,8,10]. In ornamental species such as roses, where flower production is the primary economic trait, understanding factors that influence flowering during in vitro culture may facilitate breeding, genetic transformation, physiological studies, and the rapid evaluation of flowering responses [7,8,10]. Despite its potential importance, studies investigating the influence of growth-promoting additives on in vitro flowering in *Rosa chinensis* remain limited. Therefore, evaluating both vegetative regeneration and flowering responses provides a more comprehensive assessment of the effects of these additives on plant development.

While regeneration protocols have been reported for several rose species and cultivars [11,24,25,26,27,28], no comprehensive study has systematically compared the effects of Put, PG, and CH, individually and in combination with PGRs, on both direct organogenesis and in vitro flowering of *Rosa chinensis* cv. Old Blush. Therefore, based on the reported roles of Put, CH, and PG in plant tissue culture, we hypothesized that these growth additives would differentially influence the efficiency of direct organogenesis and in vitro flowering of *Rosa chinensis* cv. Old Blush, and that their application in combination with PGRs would enhance regeneration and reproductive responses more effectively than their individual application or the control.

## 2. Materials and Methods

### 2.1. Plant Materials and Surface Sterilization

*R. chinensis* cv. Old Blush stem cuttings were collected from pot plants preserved at the herbarium of Sana Institute of Higher Education, Sari, Iran. All the leaves, thorns, and flower buds were removed from the cuttings and excised to approximately 2 cm length segments containing one axillary bud. All the explants were washed with carbendazim fungicide 1% (*w*/*v*) for 20 min to remove surface contamination agents and subsequently disinfected with sodium hypochlorite (NaOCl) 2.5% (*v*/*v*) for 15 min. Rinsing three times with autoclaved double-distilled water (DDW) with intervals of 5, 10, and 15 min was the final sterilization step before cultivation. The explants were then blotted on the sterilized filter paper to absorb extra water. Driver Kuniyuki Walnut (DKW) medium (Duchefa Biochemie, Proton Chemie Co., Ltd., Tehran, Iran) [26] was used as the basal medium for all in vitro cultures, including the establishment stage. The explants were cut to 1 cm in length and were cultured on DKW establishment medium containing 200 mg L^−1^ cefotaxime antibiotic and 50 mg L^−1^ silver nitrate (AgNO_3_) for initial establishment and selection of healthy explants for the main experiment.

### 2.2. In Vitro Direct Organogenesis

After two weeks, healthy nodal segments (fungi- and bacteria-free) were selected from the establishment stage and transferred to the direct organogenesis stage. DKW medium containing a stable concentration of 1.5 mg L^−1^ 6-Benzylaminopurine (BAP) + 0.1 mg L^−1^ 1-Naphthaleneacetic acid (NAA) [11,26,27,28] was combined with different types and concentrations of growth additives, including four levels (0, 5, 10, 20 µM) of Put, PG, and CH (0, 200, 400, 600 mg L^−1^). The selected concentrations were chosen based on our previous regeneration studies in *Rosa canina* and published reports indicating that this concentration range is effective for evaluating the physiological responses of growth additives during in vitro culture [11,24,25,26,27,28]. Therefore, the experiment consisted of four treatment groups: (i) Control without PGRs (DKW medium with without PGRs and growth additives), (ii) Control with PGRs and without growth additives (1.5 mg L^−1^ BAP + 0.1 mg L^−1^ NAA), (iii) growth additives applied individually (Put, PG, or CH in the absence of PGRs), and (iv) growth additives combined with PGRs (1.5 mg L^−1^ BAP + 0.1 mg L^−1^ NAA supplemented with Put, PG, or CH). Thus, each growth additive was evaluated both individually and in combination with PGRs to determine its independent and synergistic effects on direct organogenesis and in vitro flowering. Appropriate volumes of sterile stock solutions of Put, PG, and CH were added to the autoclaved medium after cooling to approximately 45–50 °C to obtain the final concentrations. Six to eight weeks after cultivation, the morphological (regenerated shoot number (RSN), regenerated shoot length (RSL), regenerated shoot diameter (RSD), Yellow Leaves Numbers (YLN), and green leaf percentage (GLP%)) and reproductive characteristics (flower number (FN), flower diameter (FD), flowering stem length (FSL), and flowering stem diameter (FSD)) of in vitro plantlets were evaluated.

### 2.3. In Vitro Elongation of Regenerated Shoots

Successfully regenerated shoots from the direct organogenesis step were transferred to free DKW medium without any PGRs containing 25 mg L^−1^ AgNO_3_ for elongation. The elongation step lasted for 4 weeks.

### 2.4. In Vitro Rooting and Acclimatization

Suitably sized in vitro regenerated and elongated shoots (longer than 1.5 cm) were transferred to the rooting stage containing DKW medium combined with 0.3 mg L^−1^ NAA, 0.3 mg L^−1^ indole-butyric acid (IBA), and 25 mg L^−1^ AgNO_3_ [11,26,27,28]. Subsequently, well-rooted in vitro plantlets were transferred to the acclimatization stage. An autoclaved pot mixture, including a 1:1 ratio of cocopeat: perlite, was used for transferring plantlets to the acclimation. All plantlets were maintained in the culture room for the first month of acclimatization. The acclimatized plants were then transferred to a greenhouse after one month. All regenerated shoots successfully produced roots using the previously optimized rooting medium, resulting in a rooting percentage of 100%. Following transfer to the acclimatization substrate, approximately 95% of the rooted plantlets survived and developed normally under greenhouse conditions, confirming the effectiveness of the rooting and acclimatization protocol.

### 2.5. In Vitro Culture Conditions

DKW medium was used as the basal medium for all in vitro cultures. All prepared media were solidified with 0.65% (*w*/*v*) agar and contained 3% (*w*/*v*) sucrose. Media pH was adjusted to 5.7–5.8 with 1 N NaOH or 1 N HCl before autoclaving (121 °C, 20 min). Fixed concentrations of AgNO_3_ were used in all in vitro cultures (50 mg L^−1^ for the establishment stage and 25 mg L^−1^ for direct organogenesis and rooting stages). 2 mL L^−1^ PPM was added to all media prepared in different stages and experiments. DKW medium, PGRs, Put, CH, PG, and chemicals were sourced from Duchefa Biochemie, Proton Chemie Co., Ltd., Iran. Cultures were incubated at 21 ± 2 °C under a 16 h light/8 h dark photoperiod at ~40 μmol m^−2^ s^−1^ provided by cool-white fluorescent lamps.

### 2.6. Experimental Design and Statistical Analysis

The experiment followed a completely randomized design (CRD) with three replicates per treatment and 10 explants per replicate. Data were analyzed using one-way ANOVA in SPSS v27, and treatment means were compared using Duncan’s Multiple Range Test at *p* ≤ 0.05.

## 3. Results

### 3.1. Interactions of PGRs and Growth Additives Showed Variations in R. chinensis cv. Old Blush In Vitro Morphological Traits

**Figure 1:**
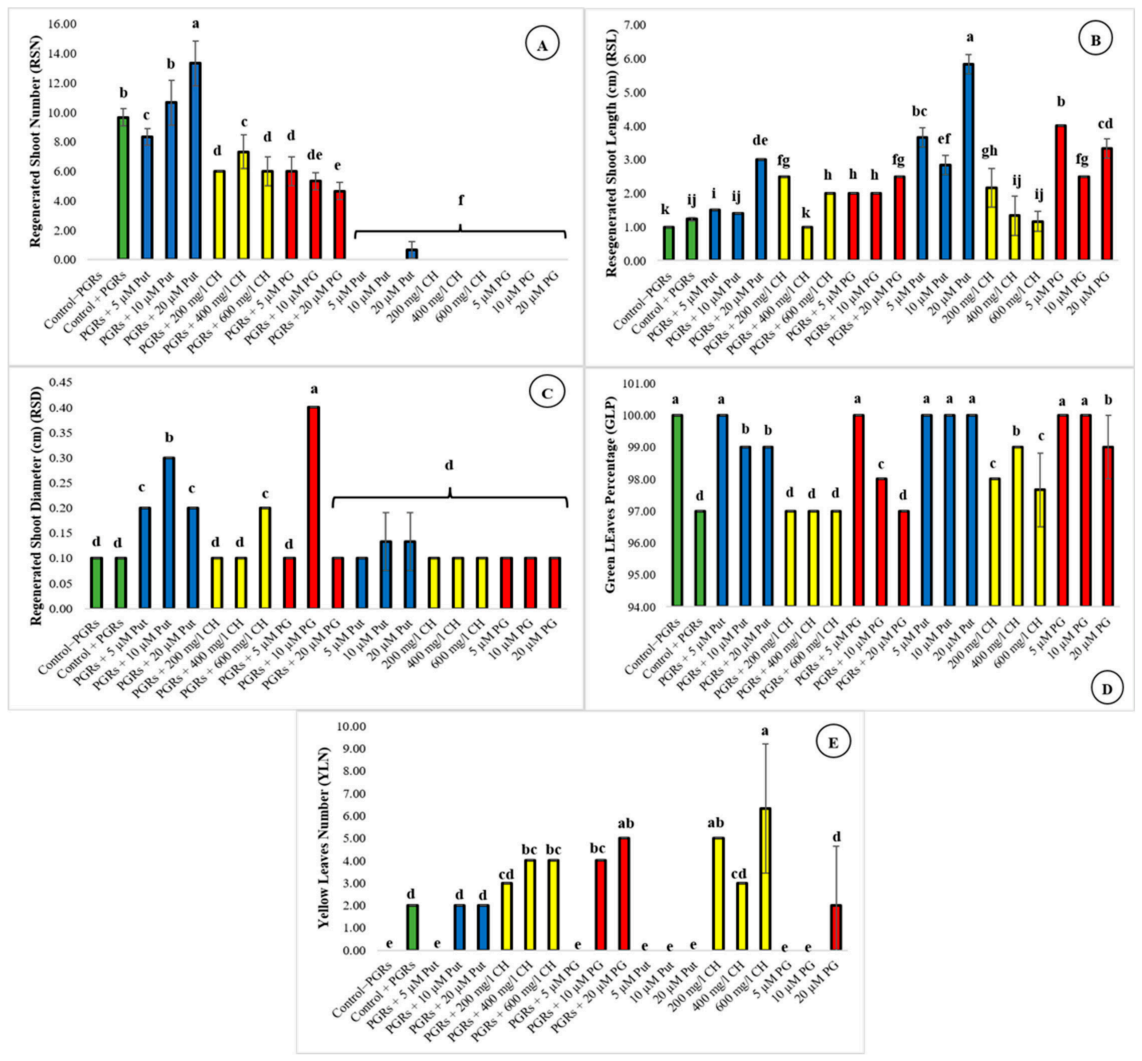
Morphological responses of explants to different treatments containing growth additives with/without PGRs during in vitro direct organogenesis ((**A**) Regenerated Shoot Number (RSN), (**B**) Regenerated Shoot Length (RSL), (**C**) Regenerated Shoot Diameter (RSD), (**D**) Green Leaves Percentage (GLP%), and (**E**) Yellow Leaves Number (YLN)) of *R. chinensis* cv. Old Blush from node explants. For improved visualization, treatment groups are distinguished by color: Control (green), Putrescine treatment groups (PGRs + Put and Put alone; blue), Casein Hydrolysate treatment groups (PGRs + CH and CH alone; yellow), and Phloroglucinol treatment groups (PGRs + PG and PG alone; red). Data are the mean  ±  standard deviation (M ± Std). Means followed by the same letter for columns (e.g., a, b, bc) are not significantly different at *p*  ≤  0.05 as determined by DMRT.

The impact of growth additives (PG, Put, and CH), both alone and in combination with PGRs, exhibited significant variations in morphological traits (*p* ≤ 0.01). The highest number of regenerated shoots was recorded when 20 µM Put was used in conjunction with PGRs (Figure 1A). A combination of 10 µM Put with PGRs and the control treatments that included PGRs ranked second, demonstrating a greater shoot regeneration rate compared to other treatments involving CH or PG. Based on the analysed results, no shoot regeneration was observed in treatments lacking PGRs (Figure 1A). Regenerated shoot length differed significantly among treatments (*p* ≤ 0.01); specifically, the exclusion of PGRs in favor of 20 µM Put yielded the greatest shoot length (Figure 1B). Conversely, control treatments using PGRs without additional growth additives produced the shortest shoots. The exogenous application of PG significantly enhanced both RSD and the GLP. The highest RSD was recorded in media supplemented with 10 µM PG and PGRs (Figure 1C), while a maximum GLP was consistently achieved in treatments containing either Put or PG (Figure 1D). In contrast, CH treatments, particularly at the 600 mg L^−1^ concentration, induced the highest rate of leaf senescence, resulting in yellow leaves per explant (Figure 1E).

### 3.2. Combinations of PGRs and Growth Additives Improved In Vitro Reproductive Characteristics in R. chinensis cv. Old Blush

**Figure 2:**
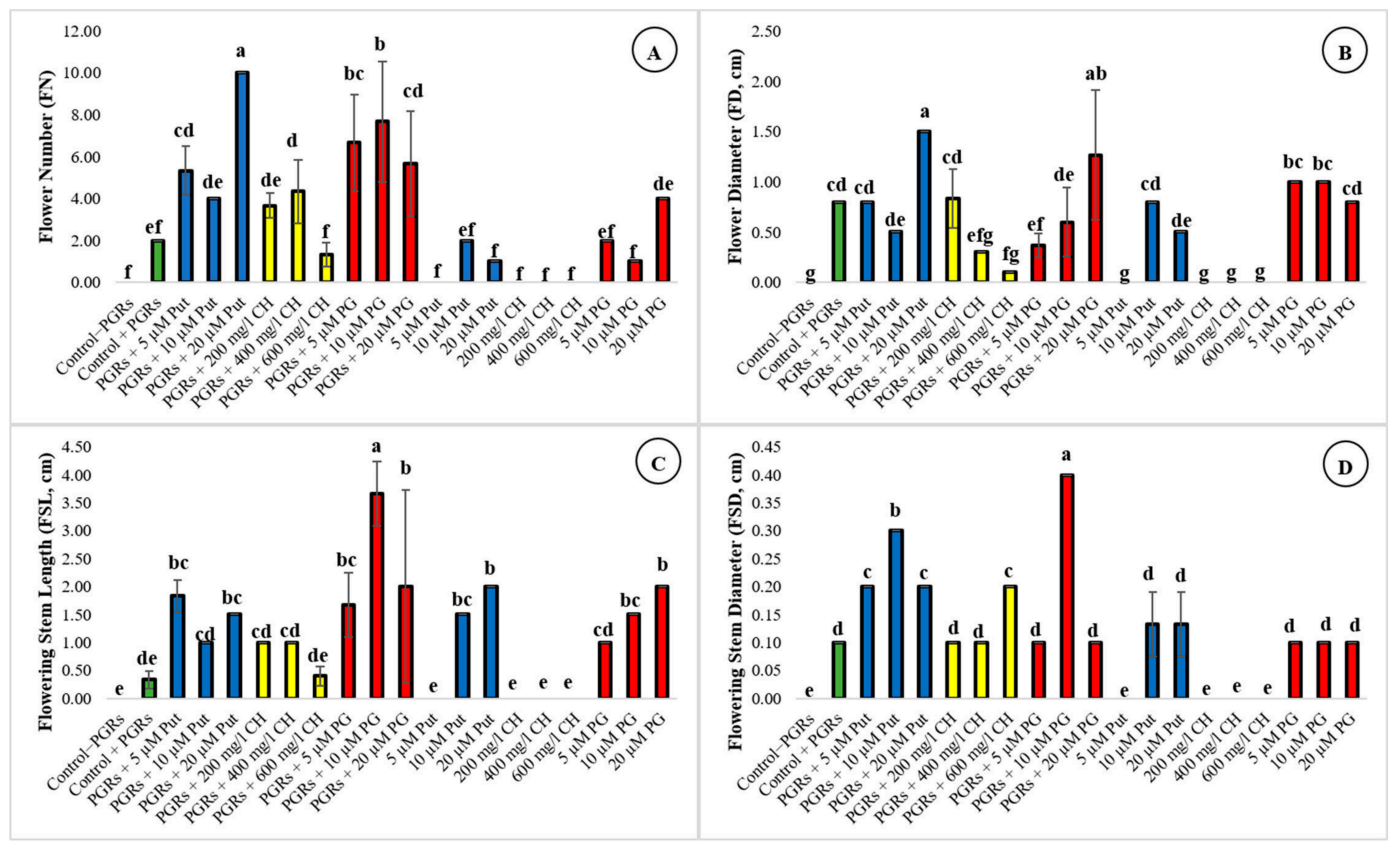
Reproductive responses of explants on different treatments containing growth additives with/without PGRs during in vitro direct organogenesis ((**A**) Flower Number (FN), (**B**) Flower Diameter (FD), (**C**) Flowering Stem Length (FSL), and (**D**) Flowering Stem Diameter (FSD)) of *R. chinensis* cv. Old Blush from node explants. For improved visualization, treatment groups are distinguished by color: Control (green), Putrescine treatment groups (PGRs + Put and Put alone; blue), Casein Hydrolysate treatment groups (PGRs + CH and CH alone; yellow), and Phloroglucinol treatment groups (PGRs + PG and PG alone; red). Data are mean  ±  standard deviation (M ± Std). Means followed by the same letter for columns (e.g., a, b, bc) are not significantly different at *p*  ≤  0.05 as determined by DMRT.

To explore the influence of growth additives and PGRs on the in vitro reproductive attributes of *R. chinensis* cv., four characteristics associated with in vitro flowering, including FN, FD, FSL, and FSD, were assessed. The analysis of the data revealed that no in vitro flowers were generated across the treatments lacking PGRs. Among the treatments that did result in in vitro flowering, notable differences were observed between them (*p* ≤ 0.01). As illustrated in Figure 2A, the highest flower yield was observed in media containing PGRs and 20 µM Put. Among the various treatments, the combination of PGRs with growth additives (specifically Put and PG) resulted in the greatest quantity of in vitro flowers (Figure 2A). In addition to achieving the maximum flower number, the flowers also exhibited a substantial diameter in the media that contained both PGRs and 20 µM Put (Figure 2B).

**Figure 3:**
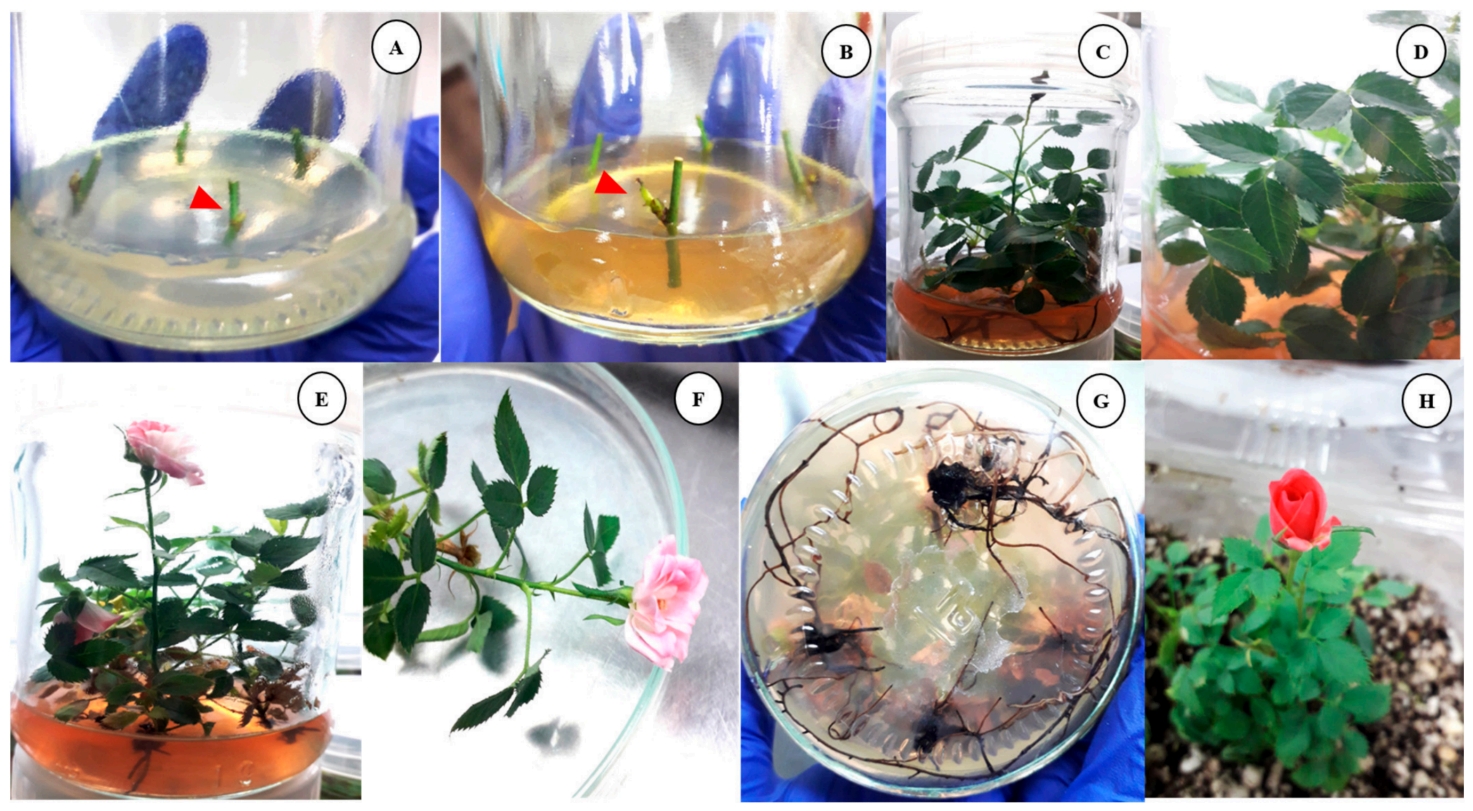
Direct organogenesis stages of *R. chinensis* cv. Old Blush from node explants. (**A**) initial node explants in DKW medium containing 50 mg L^−1^ AgNO_3_ during the establishment stage, (**B**) axillary bud growth after 2 weeks in establishment stage (media color change is due to the exposure of the media containing AgNO_3_ with light), (**C**) maximum shoot length in DKW medium containing 1.5 mg L^−1^ BAP, 0.1 mg L^−1^ NAA, 25 mg L^−1^ AgNO_3_, and 20 µM Put, (**D**) the quality of the green leaves achieved in the DKW medium containing growth additives (Put or PG), (**E**) maximum flower diameter (FD) observed in DKW medium containing 1.5 mg L^−1^ BAP, 0.1 mg L^−1^ NAA, 25 mg L^−1^ AgNO_3_, and 20 µM Put, (**F**) the quality of the flowers produced in the DKW medium containing 20 µM Put, (**G**) in vitro rooting of the plantlets, and (**H**) successfully acclimatized plant. The red arrow shows the axillary bud in each explant.

In terms of morphological traits, the greatest FSL and the highest FSD were observed in the media that included both PGRs and 10 µM PG (see Figure 2C,D). When considering both FSD and FSL, the combinations of PGRs with PG or Put yielded superior results compared to the treatments containing CH. Conversely, removing PGRs from the media and solely using growth additives did not lead to improved reproductive characteristics. Moreover, among various concentrations of PG and Put, 10–20 µM showed superior responses in combinations with PGRs to enhance morphological and reproductive regeneration efficiencies (Figure 3). 

### 3.3. Rooting and Acclimatization

All regenerated shoots successfully produced roots using the previously optimized rooting medium [11,26,27,28], resulting in a rooting percentage of 100%. Following transfer to the acclimatization substrate, approximately 95% of the rooted plantlets survived and developed normally under greenhouse conditions, confirming the effectiveness of the rooting and acclimatization protocol.

## 4. Discussion

The significant differences observed among the morphological traits indicate that the type and concentration of growth additives markedly influenced the in vitro regeneration response of *Rosa chinensis* cv. Old Blush. The highest regenerated shoot number was obtained when 20 µM Put was combined with PGRs, whereas treatments containing PG or CH, either alone or in combination with PGRs, produced comparatively lower regeneration responses. In addition, no shoot regeneration was observed in treatments lacking PGRs. These findings suggest that Put was the most effective growth additive for enhancing shoot organogenesis under the culture conditions used in this study and that growth additives alone were insufficient to induce organogenesis in the absence of PGRs. Similar observations have been reported in previous studies, where PAs were shown to improve regeneration efficiency when applied together with conventional PGRs rather than replacing them [29,30,31].

Supplementation of the culture medium with appropriate concentrations of Put and PG also improved several indicators of shoot quality. The greatest shoot elongation was observed in the treatment containing 20 µM Put, whereas the highest regenerated shoot diameter was recorded in the medium supplemented with 10 µM PG and PGRs. In addition, treatments containing Put or PG maintained a higher percentage of green leaves than the other treatments. These responses are consistent with previous reports suggesting that PAs contribute to cell division and elongation and may alleviate some of the adverse effects associated with synthetic PGRs during in vitro culture [30,32,33]. Likewise, PG has been reported to promote plant growth and improve explant quality through its involvement in physiological processes associated with plant development [34,35,36]. Although the present study did not investigate the physiological mechanisms responsible for these responses, the observed improvements are consistent with the beneficial effects of Put and PG reported in earlier studies.

In contrast, CH treatments generally resulted in lower regeneration efficiency and a greater number of yellow leaves than Put- or PG-containing media. This observation suggests that CH was less effective than the other growth additives for improving the morphological performance of *R. chinensis* under the conditions evaluated. Interestingly, this response differs from our previous findings in *Rosa canina*, where CH markedly enhanced shoot regeneration when combined with PGRs [11]. Such contrasting responses suggest that the effectiveness of CH is highly dependent on plant species and genotype, as well as the interaction between culture medium composition and endogenous physiological status. Previous studies have also reported that the response to organic additives varies considerably among plant species and tissue culture systems. Therefore, optimization of CH concentration should be considered species-specific rather than universally applicable [11,23,24,37].

The present study also demonstrated that combining growth additives with PGRs substantially improved the reproductive characteristics of *R. chinensis* cv. Old Blush. Flowering was not observed in treatments lacking PGRs, regardless of the presence of growth additives, whereas the highest flower number and flower diameter were obtained in the treatment containing PGRs supplemented with 20 µM Put. These findings suggest that PGRs were essential for floral induction under the culture conditions employed, while Put further enhanced the flowering response. Similar positive effects of PAs on floral development have been reported in several plant species [37,38,39]. Previous studies have suggested that PAs may participate in developmental signaling pathways and interact with endogenous plant hormones during reproductive development [39]. Although these mechanisms were not investigated in the present study, they may partially explain the improved flowering observed following Put application.

Among the evaluated growth additives, PG produced the greatest flowering stem length and flowering stem diameter when combined with PGRs, indicating its beneficial effect on flower quality traits. Previous studies have suggested that PG may improve structural development and reduce physiological disorders during in vitro culture, which could contribute to stronger flower stalks and improved plant quality [37,38,39,40,41,42]. By comparison, CH consistently produced lower reproductive responses than either Put or PG. Under the experimental conditions of this study, Put and PG therefore appeared to be more effective than CH in enhancing both vegetative regeneration and in vitro flowering.

Although the present study demonstrated the beneficial effects of Put and PG on direct organogenesis and in vitro flowering of *R. chinensis* cv. Old Blush, several limitations should be acknowledged. The study focused on morphological and reproductive responses under in vitro conditions and did not include physiological, biochemical, or molecular analyses to verify the mechanisms underlying the observed responses. Therefore, the proposed roles of Put, PG, and CH should be regarded as possible explanations based on previous literature rather than direct evidence from the present investigation. Furthermore, only a single rose cultivar and selected concentration ranges were evaluated, which may limit the broader applicability of the findings to other genotypes or culture conditions. Future studies incorporating endogenous hormone analysis, PA metabolism, oxidative stress markers, gene expression profiling, and additional rose cultivars would provide a more comprehensive understanding of the mechanisms by which these growth additives regulate in vitro regeneration and flowering.

## 5. Conclusions

Our study reveals that the in vitro regeneration success of *R. chinensis* cv. Old Blush (both in morphological and reproductive aspects) requires a synergistic interaction between PGRs and specific growth additives, with 20 µM Put and 10 µM PG maximizing morphological and reproductive yield. These findings, highlighting the superior efficacy of targeted signaling molecules over general organic nitrogen, establish a refined protocol for efficient in vitro regeneration and flowering in controlled environments.

## Data Availability

Data will be made available on request.
